# Prevalence and factors associated with undernutrition among children under the age of five years in Benin

**DOI:** 10.1371/journal.pone.0289933

**Published:** 2023-08-10

**Authors:** Isaac Yeboah Addo, Elijah Frimpong Boadu, Emmanuel Osei Bonsu, Caleb Boadi, Frederick Asankom Dadzie

**Affiliations:** 1 Centre for Social Research in Health, University of New South Wales, Sydney, Australia; 2 School of Built Environment, University of New South Wales, Sydney, Australia; 3 Department of Epidemiology and Biostatistics, Kwame Nkrumah University of Science and Technology, Kumasi, Ghana; 4 Department of Operations and Management Information Systems, University of Ghana, Accra, Ghana; 5 Centre for Ecosystem Science, School of Biological, Earth and Environmental Sciences, University of New South Wales, Sydney, Australia; 6 Evolution and Ecology Research Centre, School of Biological, Earth and Environmental Sciences, University of New South Wales, Sydney, Australia; National University of Sciences and Technology, PAKISTAN

## Abstract

**Background:**

Benin ranks as one of the countries in the world with an alarmingly high prevalence of stunting, wasting, and underweight in children under five years. However, limited studies have examined the factors associated with the prevalence of these undernutrition indicators among children under five years in the country. This study aimed to fill this research gap by examining the prevalence rates and factors associated with stunting, wasting, and underweight among this specific population of interest.

**Methods:**

This quantitative study utilised data from the most recent Benin Demographic and Health Survey (BDHS) conducted in 2017–18. The survey employed a nationally representative cross-sectional design and utilised a two-stage stratified cluster sampling technique to select participants. The study included a sample of 13,589 children under the age of five years. The main analytical approach employed was binary logistic regression, which was used to explore the associations between undernutrition (the combined outcome variable representing stunting, wasting, and underweight) and various socio-demographic factors.

**Results:**

The combined prevalence of stunting, wasting, and underweight among children under five years in Benin during the 2017–18 survey period was 14.95%. Several factors were significantly associated with these indicators of undernutrition, including female gender (AOR = 0.71, 95% CI = 0.59–0.85), birth weight of 4.1 kg and over (AOR = 0.26, 95% CI = 0.14–0.48), multiple births (AOR = 3.22, 95% CI = 2.11–4.91), and a child’s experience of diarrhoea (AOR = 1.76, 95% CI = 1.40–2.20). Furthermore, the prevalence of these undernutrition indicators was higher among children whose mothers had lower levels of education (AOR = 0.82, 95% CI = 0.01–0.42) and were unmarried (AOR = 0.67, 95% CI = 0.49–0.93).

**Conclusions:**

This present study confirms that undernutrition rates are elevated in Benin and are closely linked to perinatal factors such as birth weights and multiple births, postnatal health conditions including diarrheal episodes, and socio-demographic determinants such as a child’s gender, maternal education level, and marital status. Therefore, there is the need to consider specific modifiable factors, such as low birth weight, episodes of child diarrhoea, and maternal education as priority targets for child nutrition interventions in Benin.

## Introduction

Globally, stunting, wasting and underweight which are common forms of undernutrition remain a significant public health challenge, particularly, in children aged under 5 years [1]. In 2020, the World Health Organisation estimated that among children under the age of 5 years, nearly 149 million were stunted, 85 million were underweight and 45 million were wasted [2, 3]. This presents a significant health challenge given that undernutrition in children raises their risk of infection and causes poor or delayed cognitive development [4]. Additionally, undernourished children are more likely to die of diseases such as respiratory infections and malaria as they have decreased biological capacity to fight infections [5]. These issues suggest the need for urgent strategies toward decreasing undernutrition in children.

Although the burden of undernutrition in children under 5 years of age is present in 124 countries worldwide, the highest prevalence of the various forms of undernutrition has been observed in children living in African and Asian countries [6]. A report by the WHO, UNICEF and the World Bank in 2012 estimated that out of 165 million children under 5 years who were stunted worldwide, over one-third resided in Africa [7]. In their 2020 report, UNICEF, the WHO and the World Bank stated that there has been a global decline in the number of children with stunting in all regions of the world except Africa [8]. Several country-specific studies in the context of Africa have also revealed a high prevalence of stunting, underweight and wasting. For example, Madiba, Chelule [9], Yirga, Melesse [10] and Anin, Saaka [11], found that the prevalence of all forms of undernutrition in South Africa, Ethiopia and Ghana respectively, has been over the considerable threshold limits of 30% stunting, 15% wasting, and 10% underweight. The high prevalence of undernutrition in African countries underscores the importance of prioritising comprehensive and context-specific strategies to improve child nutrition, healthcare, and socio-economic development. Collaborative efforts involving governments, international organisations, and communities are essential in combating undernutrition and ensuring the healthy growth and development of children across the African continent.

Studies have identified various factors that play a significant role in contributing to the elevated prevalence of undernutrition among children during their early years. Child-related factors, including age, gender, body size at birth, birth spacing, vaccination status, and birth order, have been found to have an impact on nutritional outcomes [9, 11–15]. Additionally, maternal characteristics, such as level of education, age at childbirth, utilisation of antenatal care (ANC) services, place of residence, wealth index, body mass index (BMI), quality of water used, hygiene and sanitation practices, and family size, have also been identified as influential factors in child undernutrition [9, 11–15]. These factors collectively contribute to the complex web of determinants that influence the nutritional status of children, emphasising the multifaceted nature of undernutrition and the need for comprehensive interventions that address these various factors simultaneously.

Although factors contributing to the prevalence of childhood undernutrition have been a subject of research in recent years, evidence shows variations in the specific factors contributing to undernutrition across countries and regions. For instance, studies in Senegal and Tanzania found that the gender of a child is a significant indicator of undernutrition, noting that boys are more likely to be stunted than girls [16, 17]. Contrary to this, however, other studies, e.g. Ndiku, Jaceldo-Siegl [18] in Kenya showed that girls were more likely to be stunted, underweight and wasted than boys. Furthermore, Amadu, Seidu [19] and Moshi, Sebastian [17], found that children aged between 18–23 months were more likely to be stunted, underweight and wasted than children of other age groups. In contrast, other studies indicate that among children under 5 years of age, those in the age group of 36–47 months were significantly more likely to be stunted, underweight and wasted [20, 21]. These findings suggest that factors associated with undernutrition may differ from country to country. Considering that Benin is recognised as the country in Sub-Saharan Africa (SSA) with the highest prevalence of stunting, underweight, and wasting among under 5 children [19], we sought to contribute to the current literature by examining the combined prevalence of stunting, underweight and wasting in Benin and the factors associated with these three undernutrition indicators. This study is timely considering that no nationally representative study, to the best of our knowledge, has examined the factors contributing to the high undernutrition status among children under 5 years of age in Benin. The outcomes are critical to the development of tailored interventions for reducing undernutrition in the country and even beyond.

## Methods

### Study design

This is a nationally representative cross-sectional study which took place in Benin and employed household interviews through administered questionnaires. Data used for the study were based on the 2017–2018 Benin Demographic and Health Survey [22].

### Study population

This study was designed as a household survey aimed at interviewing all caregivers with children under the age of 5 years.

### Data source

The Benin Demographic and Health Survey (BDHS) is a comprehensive survey conducted at the national level, designed to collect current and reliable health information for monitoring and evaluating the health situation in Benin. The survey data is managed by key institutions including the Ministry of Planning and Development and the National Institute of Statistics and Economic Analysis (INSAE). These institutions work in collaboration with other stakeholders, such as the Ministry of Health, the Centre for Parasitology and Cardiology Laboratory of the National Hospital, and the Permanent Secretariat of the Food and Nutrition Council [23]. ICF International provided technical support when conducting the survey whereas UNAIDS, UNICEF, UFPA and the World Bank provided funding support [22, 23].

The BDHS plays a crucial role in providing policymakers, researchers, and health practitioners with valuable data and insights into various health indicators, including maternal and child health, nutrition, reproductive health, and infectious diseases. By ensuring the representativeness of the sample and employing rigorous data collection methods, the BDHS contributes to the generation of high-quality and reliable health information that informs evidence-based decision-making and policy formulation in Benin [22, 23].

The involvement of multiple institutions and stakeholders in the management of the BDHS demonstrates a collaborative and multidisciplinary approach to data collection, analysis, and dissemination. This collaboration enhances the credibility and reliability of the survey findings and strengthens the capacity for monitoring and improving the health and well-being of the population in Benin [22, 23].

### Sampling

The 2017–2018 BDHS report [22] provides a detailed description of the sampling process. A two-stage stratified cluster sampling technique was used to select participants for the survey. In the first stage, a probability-proportional to size (PPS) technique was used to select an enumeration area, which consisted of a large cluster of households. A sample frame was developed by listing houses in each enumeration area before sampling. In the second stage, households were selected from each enumeration area to ensure representativeness [22]. The final dataset comprises a total of 14,156 households, 15,928 women in their reproductive age groups (15–49 years), 7,595 men aged 15–59, and 13,589 and 13,643 unweighted and weighted children under 5 years of age, respectively [22].

### Variable selection and measurement

The study included 13,589 children under 5 years drawn from the children’s file (BJKR71DT.ZIP) in the dataset. Undernutrition was computed using underweight (weight for age in z-scores), stunting (height for age in z-scores) and wasting (weight for height in z-scores) as specified in the Demographic and Health Survey (DHS) and based on WHO child growth standards [24]. As shown in Table 1, children under five years of age with a z-score less than -2 standard deviation were categorised as stunted, wasted, and underweight. The outcome variables were categorised into two: 1) undernutrition (combination of stunting, wasting, and underweight) which was coded as 1 and 2) normal nutrition which was coded as 0 (Table 1).

**Table 1:** Description and categorisation of variables used in the analysis.

Variables	Codes used for the analysis
*Outcome*	
Stunting (Height for age index)	0 = Normal height for age/Not stunted (HAZ -2SD and above), 1 = Stunted (HAZ<-2 SD)
Wasting (Weight for height index)	0 = Normal weight for height/Not wasted (WHZ -2SD to +2SD), 1 = Wasted (WJZ <-2 SD)
Underweight (Weight for age index)	0 = Normal weight for age/Not underweight (WAZ-2SD and above), 1 = Underweight (WAZ <-2SD)
*Independent*	
Age of Child	1 = 0–18 months, 2 = 19–37 months, 3 = 38+ months
Sex of Child	1 = Male, 2 = Female
Birth Weight	1 = <2.5 kg, 2 = 2.5–4.0 kg, 3 = 4.1+ kg
Birth type	1 = Single, 2 = Multiple
Birth Order	1 = 1^st^ - 4^th^ child, 2 = 5^th^ - 8^th^ child, 3 = 9^th^ child and over
Number of under 5 children in the household	1 = 0–4, 2 = 5–9, 3 = 10+
Child diarrhoea	0 = No, 1 = Yes
Months of Breastfeeding	1 = 0–18 months, 2 = 19–37 months, 3 = 38 months or more
Maternal Age at First Birth	1 = 11–19 years, 2 = 20–28 years, 3 = 29+ years
Education Level	0 = No formal education, 1 = Primary level education, 2 = Secondary level education, 3 = Higher level education
Place of Residence	1 = Urban, 2 = Rural
Wealth Index	1 = poor, 2 = Middle, 3 = Rich
Marital Status	1 = Not married, 2 = Married
Nutritional counselling during pregnancy	0 = No, 1 = Yes
Toilet Facility	1 = Improved, 2 = Unimproved
Source of Water	1 = Improved water, 2 = Unimproved water
Electricity access	0 = No, 1 = Yes
Access to Media	0 = No, 1 = Yes
Type of cooking fuel	1 = Improved, 2 = Unimproved
Antenatal visit during pregnancy	0 = No, 1 = Yes
Postnatal Visit	0 = No, 1 = Yes
Current work status	1 = Not working, 2 = Working

Based on theoretical and empirical literature [9, 11–15], we identified a total of twenty-two (22) variables as possible risk factors for undernutrition in children under 5 years in Benin. These included child and maternal factors, namely age of child, sex or gender of child, birth weight, birth type, birth order, number of children under five years of age in a household, child diarrhoea, breastfeeding status, maternal age at first birth, educational level, place of residence, wealth index, marital status, nutritional counselling during pregnancy, type of toilet facility, source of drinking water, access to electricity, access to media, type of cooking fuel, antenatal visits, postnatal visits, and current work status (Table 1).

In this study, water sources were classified as improved and unimproved, drawing on the research conducted by [25, 26]. Improved water sources included piped water in dwellings, piped water to yard/plot, public tap/standpipe, tube well or borehole, protected well, protected spring, and bottled water. In contrast, water sources such as unprotected wells, rivers/dams/lakes/ponds/streams/canals/irrigation systems, tanker trucks, and carts with small tanks were classified as unimproved due to their higher susceptibility to contamination from various organisms. Cooking fuel types were also classified into Improved and Unimproved categories. Improved cooking fuels encompassed modern fuels known for their enhanced safety and efficiency. This category includes liquefied petroleum gas (LPG), natural gas, electricity, as well as clean fuels like ethanol or biogas. On the other hand, Unimproved cooking fuels consisted of unconventional and less efficient options that may pose higher health risks. Examples include solid fuels such as firewood, coal, charcoal, agricultural residues, and traditional biomass [27, 28]. Furthermore, access to electricity was considered as a potential determinant of undernutrition. Electricity has multiple pathways through which it can impact undernutrition. Firstly, it enables households to utilise refrigeration and other electrical appliances for proper food storage, reducing spoilage and maintaining food safety and hygiene [29]. Additionally, electricity plays a crucial role in ensuring access to improved water through electric-powered pumps for water supply systems, which is essential for maintaining food hygiene and preventing waterborne diseases [30].

### Data analysis

Stata/SE version 14 software was used for the data analysis. The data were set for survey analysis using svyset command. Samples were weighted based on recommendations in the Demographic and Health Survey (DHS) dataset [31]. The significance level was set at 0.05, with a corresponding confidence level of 95%. To check for multicollinearity, Variance Inflation factor (VIF) was calculated for each explanatory variable. The mean VIF was 3.25. VIF greater than 10 was considered an indication of multicollinearity [32]. Additionally, a correlation matrix for the variables was developed to identify highly correlated variables. To ensure the data fit the model well, diagnostic tests such as the goodness of fit test were conducted which produced a p-value of 0.19 indicating the data fits the model [33]. To estimate the prevalence of undernutrition in Benin, we developed a single outcome variable based on the amalgamation of the three anthropometric measures—underweight (weight for age in z-scores), stunting (height for age in z-scores) and wasting (weight for height in z-scores). Our approach was guided by a previous study and the outcome was used as our indicator of undernutrition in Benin [19]. Binary logistic regression was used to explore the associations between the outcome variable and the multiple risk factors.

### Ethical consideration

The data for this study were collected in adherence to participants’ privacy and confidentiality. ICF International confirms that the survey conforms to the U.S. Department of Health and Human Services rules for respecting human subjects’ rights. No additional consent was required for this study as the data is secondary, publicly available and can be accessed in the public domain. The data is available at https://dhsprogram.com/data/available-datasets.cfm) [22].

## Results

### Socio-demographic characteristics of respondents

The sample of children under 5 years old was evenly distributed across three age categories: 0–18 months (34%), 19–37 months (30%), and 38 months or more (36%). Among the sample, 51% were male children, while the remaining 49% were female. The majority of children (85%) had birth weights ranging between 2.5 and 4.0 kilograms, with a small proportion (4%) weighing more than 4.1 kilograms. Single births accounted for 95% of all births, while multiple births accounted for only 5%. Most children (71%) were within the 1–4 birth order range, whereas a small percentage (3%) fell into the 9+ range. Within households, the majority of children (92%) were between 0–4 years old (Table 2). Regarding recent health status, approximately 89% of the children did not experience diarrhoea in the preceding two weeks, while 11% had diarrhoea.

**Table 2:** Socio-demographic characteristics of children under 5 and their mothers in Benin.

Variable	N = 13,589	Weighted %
	(%)	(CI)
**Age of Child (months)**		
0–18 months	4, 577 (33.68)	33.60 (33.21–34.63)
19–37 months	4,111 (30.25)	30.42 (29.89–31.13)
38+ months	4,901 (36.07)	35.98 (34.85–37.07)
**Sex of Child**		
Male	6,910 (50.85)	50.99 (50.29–52.04)
Female	6,679 (49.15)	49.01 (48.25–50.19)
**Birth Weight**		
< 2.5 kg	960 (11.70)	11.60 (11.21–13.38)
2.5–4.0 kg	6,923 (84.38)	84.60 (84.37–86.27)
4.1 + kg	322 (3.92)	3.80 (3.01–4.08)
**Birth Type**		
Single birth	12,899 (94.92)	94.70 (93.79–95.05)
Multiple births	690 (5.08)	5.30 (4.89–6.10)
**Birth Order**		
1–4	9,633 (71.11)	71.19 (70.85–72.23)
5–8	3,492 (25.70)	25.76 (24.98–27.14)
9 +	434 (3.19)	3.05 (2.98–4.04)
**Number of Under 5 Children in the Household**		
0–4	12,468 (91.75)	91.76 (90.85–93.23)
5–9	1,060 (7.80)	7.74 (6.97–9.03)
10 +	61 (0.45)	0.50 (0.00–0.10)
**Child Diarrhoea**		
No	11,170 (89.27)	89.48 (88.89–90.11)
Yes	1,342 (10.73)	10.52 (10.14–11.29)
**Months of Breastfeeding**		
0–18 months	3,945 (85.06)	85.23 (84.27–86.00)
19–37 months	626 (13.50)	13.41 (12–15)
38 + months	67 (1.44)	1.36 (0.98–2.32)
**Maternal Age at First Birth**		
11–19	7,095 (52.21)	52.26 (51.02–54.08)
20–28	6,171 (45.41)	45.48 (44.50–47.06)
29 +	323 (2.38)	2.26 (1.98–3.22)
**Education Level**		
No education	8,036 (65.76)	65.71 (64.56–68.08)
Primary	2,447 (18.01)	18.22 (17.98–19.06)
Secondary	2,010 (14.79)	14.76 (13.87–16.28)
Higher	196 (1.44)	1.31 (1.06–2.33)
**Place of Residence**		
Urban	5,401 (39.75)	38.53 (36.75–41.54)
Rural	8,188 (60.25)	61.47 (59–64)
**Wealth**		
Poor	5,772 (42.48)	41.70 (38.98–45.38)
Middle	2,725 (20.05)	20.26 (19.27–22.24)
Rich	5,092 (37.47)	38.04 (35.31–41.04)
**Marital Status**		
Not married	822 (6.05)	6.26 (5.98–6.65)
Married	12,767 (93.95)	93.74 (92.79–94.04)
**Nutritional Counselling during Pregnancy**		
No	3,758 (47.19)	46.67 (45.88–49.26)
Yes	4,206 (52.81)	53.33 (51.00–55.23)
**Toilet Facility**		
Improved	3,667 (27.47)	27.42 (25.79–30.34)
Not Improved	9,682 (72.53)	72.58 (70.05–74.39)
**Source of Water**		
Improved water	8,911 (66.75)	66.05 (64.86–68.89)
Unimproved water	4,438 (33.25)	33.95 (32.64–36.28)
**Electricity Access**		
No	9,094 (68.12)	68.13 (66.78–71.19)
Yes	4,255 (31.88)	31.87 (29.64–34.12)
**Access to Media**		
No	5,936 (44.47)	44.41 (42.89–46.23)
Yes	7,413 (55.53)	55.59 (54.24–58.00)
**Type of Cooking Fuel**		
Improved	4,27 (3.20)	2.88 (2.27–3.38)
Unimproved	12,922 (96.80)	97.12 (96.89–98.12)
**Antenatal Visit during Pregnancy**		
No	1,030 (11.75)	11.19 (9.25–12.89)
Yes	7,736 (88.25)	88.81 (88.12–91.29)
**Postnatal Visit**		
No	7,174 (80.11)	80.78 (79.20–82.08)
Yes	1,781 (19.89)	19.22 (18.11–21.09)
**Current Work Status**		
Not working	2,609 (19.20)	19.14 (18.28–21.09)
Working	10,980 (80.80)	80.86 (79.23–82.81)

Note: N represents the total count of individuals given in percentages and CI is the weighted confidence interval

In terms of maternal characteristics, slightly more than half (52%) of the mothers were adolescents (11–19 years old) at the time of their first birth, with only 2% being 29 years or older. The majority of the mothers (66%) had no formal education, while only 1% had a higher level of education. Most mothers (61%) resided in rural areas, while a little over one-third (39%) lived in urban areas. A significant proportion of the mothers (42%) belonged to the poor wealth category, while 38% were classified as rich (Table 2).

The majority of the mothers (94%) were married, while the remaining 6% were unmarried. A majority of the mothers (66%) had access to improved drinking water, and more than half (53%) received nutritional counselling during pregnancy. Approximately 89% of the mothers attended antenatal clinics during pregnancy, while only 19% visited the post-natal clinic (Table 2).

### Prevalence of stunting, wasting and underweight among children under 5 in Benin

The overall prevalence of undernutrition in Benin, as indicated by stunting, wasting, and underweight, was approximately 15%. Among the three indicators, stunting had a prevalence of 31.56%, wasting had a prevalence of 5.04%, and underweight had a prevalence of 16.65%. Analysing the data by age groups, the highest prevalence of undernutrition (35.7%) was observed among children aged 38 months and above. Among specific indicators, the highest prevalence of stunting (39.2%) was found among children aged 19–37 months, while the highest prevalence of wasting (56.8%) and underweight (36.6%) was observed among children aged 0–18 months.

Regarding gender differences, male children exhibited a higher prevalence of undernutrition (54.4%) compared to female children. Furthermore, when considering specific indicators, male children had a higher prevalence of stunting only (55.1%), wasting only (58.0%), and underweight only (53.6%) compared to female children. The majority of undernourished children (74.3%) had birth weights ranging between 2.5 and 4.0 kg.

Examining the association with birth characteristics, single births had a higher prevalence of undernutrition (89.8%), and mothers aged 11–19 years showed a higher prevalence (55.3%) compared to other age groups. Maternal education played a role, with a higher prevalence of undernutrition observed among mothers with no formal education (70.5%) compared to those with higher education levels. Geographically, undernutrition was more prevalent among children residing in rural areas (65.9%) compared to urban areas (Table 3).

**Table 3:** Prevalence of stunting, wasting and underweight (undernutrition).

Variable	Weighted %			
	Overall prevalence (CI)	Stunting (CI)	Wasting (CI)	Underweight (CI)
**Total**	**14.95 (13.98–16.26**)	**31.56 (30.89–32.24)**	**5.04 (4.96–6.54)**	**16.65 (15.68–17.85)**
**Age of Child (months)**				
0–18 months	35.69 (33.54–38.29)	25.27 (23.59–27.21)	56.84 (52.86–61.28)	36.57 (34.23–39.89)
19–37 months	32.52 (30.34–35.56)	39.16 (37.68–41.53)	23.82 (21.64–27.83)	31.49 (29–33)
38+ months	31.79 (30.27–34.35)	35.57 (34.28–37.45)	19.35 (16.23–23.46)	31.93 (30.39–34.48)
**Sex of Child**				
Male	54.45 (52.56–57.29)	55.13 (53.59–57.48)	58.00 (54.76–62.26)	53.63 (51.64–56.28)
Female	45.55 (43.87–48.74)	44.87 (43.56–47.87)	42.0 (38.42–46.47)	46.37 (44.73–49.28)
**Birth Weight**				
< 2.5 kg	23.80 (21.45–27.38)	17.96 (16.68–20.10)	21.26 (17.19–26.43)	23.00 (20.67–26.12)
2.5–4.0 kg	74.28 (71.24–77.85)	79.36 (77.28–81.31)	77.22 (72.48–81)	74.80 (72.32–78.15)
4.1 + kg	1.92 (1.64–3.22)	2.68 (1.98–3.68)	1.52 (1.11–3.42)	2.20 (1.89–3.23)
**Birth Type**				
Single birth	89.82 (88.78–92.43)	92.48 (91.65–94.37)	90.06 (86.28–93.31)	89.86 (88.25–92.26)
Multiple birth	10.17 (8.95–12.16)	7.52 (6.64–9.23)	9.94 (7.27–13.65)	10.14 (8.93–12.71)
**Birth Order**				
1–4	70.20 (68.25–73.31)	69.37 (68.15–71.23)	70.08 (66.80–74.07)	70.76 (68.07–73.24)
5–8	27.02 (25.18–30.23)	26.28 (0.25–0.29)	26.96 (23.44–31.24)	26.43 (23.65–29.02)
9 1	2.78 (2.15–4.15)	3.64 (3.33–4.22)	2.96 (2.10–5.25)	2.82 (2.19–4.07)
**Child Diarrhoea**				
No	85.72 (84.23–87.48)	87.91 (87.11–89.24)	81.09 (77.26–84.32)	85.51 (84–87)
Yes	14.28 (13.16–16.78)	12.09 (11.87–13.33)	18.91 (16.27–23.87)	14.49 (13.23–16.33)
**Months of Breastfeeding**				
0–18 months	77.63 (74.87–81.12)	73.13 (70.28–76.78)	86.03 (82.41–89.04)	79.12 (76.18–82.18)
19–37 months	20.69 (18.38–24.22)	24.54 (21.99–28.54)	13.19 (10.15–17.67)	19.22 (16.12–22.19)
38+ months	1.68 (1.21–3.53)	2.34 (2.04–4.07)	0.77 (0.04–3.89)	1.66 (1.22–3.02)
**Number of Children in Household**				
0–4	89.25 (86.29–92.65)	90.41 (88.85–92.07)	90.32 (87.56–93.22)	89.60 (87.28–92.54)
5–9	9.39 (7.75–12.03)	8.78 (7.29–11.28)	7.81 (6.84–11.22)	9.19 (7.28–12.45)
10 +	1.36 (0.00–4.03)	0.81(0.00–2.08)	1.61(1.33–5.12)	1.21(0.00–3.82)
**Maternal Age at First Birth**				
11–19	55.31 (52.85–58.43)	55.58 (53.64–58.78)	49.88 (46.26–54.36)	55.35 (53.12–58.43)
20–28	42.63 (40.23–45.63)	42.44 (40.27–45.46)	48.32 (44.36–53.74)	42.58 (40.65–45.78)
29 +	2.06 (1.64–3.34)	1.98 (1.23–2.50)	1.81 (1.22–3.54)	2.05 (1.62–3.05)
**Education Level**				
Primary	18.22 (17.17–19.19)	17.07 (15.18–19.47)	15.89 (13.36–19.65)	18.04 (16.53–20.36)
No education	65.71 (64.18–68.17)	71.17 (0.68–0.74)	67.37 (63.13–72.81)	69.76 (67.15–73.93)
Tertiary	16.07 (15.15–17.75)	11.76 (0.10–0.13)	16.74 (13.37–21.47)	12.20 (10.23–14.56)
**Place of Residence**				
Urban	34.08 (31.12–37.14)	33.65 (30.19–36.64)	40.09 (36.22–44.46)	34.64 (31.67–38.29)
Rural	65.92 (63.18–69.16)	66.35 (63.26–69.96)	59.91 (56.76–64.74)	65.36 (62.74–69.56)
**Wealth**				
Poor	32.68 (29.21–36.16)	48.69 (45.73–52.65)	43.22 (38.46–49.33)	48.40 (45.26–52.25)
Middle	49.00 (45.94–53.43)	20.51 (19.18–23.23)	18.41 (15.64–23.56)	18.30 (16.32–21.45)
Rich	18.32 (16.67–21.44)	30.80 (27.79–34.11)	38.37 (33.87–44.74	33.30 (30.63–37.81)
**Nutritional counselling during pregnancy**				
No	49.53 (.46.42–53.35)	49.44 (47.84–53.47)	45.29 (40.53–51.67)	48.16 (45.67–52.23)
Yes	50.47 (47.54–54.76)	50.56 (48.46–53.23)	54.71 (49.15–60.74)	51.84 (48.27–55.13)
**Marital Status**				
Not married	6.96 (6.27–9.65)	5.99 (5.08–7.00)	8.69 (6.89–12.01)	6.85 (6.26–8.12)
Married	93.04 (91.22–94.18)	94.01 (93.36–95.17)	91.31 (88–94)	93.15 (92.39–94.14)
**Toilet Facility**				
Improved	22.59 (20.29–26.50)	21.35 (19.18–24.23)	26.69 (23.95–31.45)	23.26 (20.32–26.15)
Not Improved	77.41 (74.98–80.74)	78.65 (76.68–81.56)	73.30 (69.23–77.96)	76.74 (73.51–80.36)
**Source of Water**				
Improved water	63.55 (60.36–67.15)	62.40 (59.85–66.32)	64.84 (60.96–70.33)	64.37 (61.29–68.33)
Unimproved water	36.45 (33.42–40.14)	37.60 (34.46–41.85)	35.15 (30.20–40.68)	35.63 (32.69–39.84)
**Electricity Access**				
No	73.94 (70.74–77.16)	75.19 (73.14–78.18)	65.91 (60.34–71.21)	73.29 (70.16–76.18)
Yes	26.06 (23.78–30.21)	24.81 (22.19–27.56)	34.09 (29.65–40.56)	26.71 (0.24–0.30)
**Access to Media**				
No	48.65 (45.40–52.60)	49.00 (43.03–52.90)	47.18 (42.19–52.14)	48.14 (43.54–52.23)
Yes	51.35 (48.13–55.85)	51.00 (48.48–54.04)	52.82 (48.21–58.47)	51.86 (48.56–55.82)
**Type of Cooking Fuel**				
Improved	1.54 (1.19–2.13)	1.31 (1.23–2.44)	0.02 (0.01–0.04)	1.62 (1.22–2.52)
Unimproved	98.46 (98.82–99.17)	98.69 (98.43–99.21)	97.70 (96.54–99.20)	98.38 (98.06–99.31)
**Antenatal Visit during Pregnancy**				
No	15.77 (13.51–19.63)	14.00 (0.12.48–17.25)	15.77 (12.81–20.63)	15.89 (13.37–19.46)
Yes	84.23 (0.81–0.87)	86.00 (0.83–0.88)	84.23 (80.65–88.21)	84.11 (81.91–87.18)
**Postnatal Visit**				
No	80.64 (77.83–84.35)	81.93 (80.96–84.18)	79.35 (75.89–83.53)	80.31 (77.66–83.88)
Yes	19.36 (16.56–23.47)	18.07 (16.78–20.24)	20.65 (17.68–25.13)	19.69 (17.48–23.38)
**Current Work Status**				
Not working	19.77 (17.68–22.64)	19.00 (17.84–21.47)	20.23 (17.89–24.56)	19.66 (17.27–22.85)
Working	80.23 (77.63–83.37)	81.00 (79.38–83.23)	79.77 (76.28–83.36)	80.34 (78.45–83.27)

Note: CI = Confidence Interval

### Associations between undernutrition and socio-demographic factors

Table 4 presents significant associations between the undernutrition indicators and various factors. Female children under five years of age exhibited 0.71 lower odds of experiencing stunting, wasting, and underweight compared to their male counterparts (AOR = 0.71, 95% CI = 0.59–0.85). Birth weight showed a significant association, with children weighing between 2.5–4.0kg having 0.38 odds of undernutrition compared to those with a birth weight below 2kg (AOR = 0.38, 95% CI = 0.30–0.50). Conversely, children with a birth weight above 4kg had 0.26 lower odds of experiencing stunting, wasting, and underweight compared to those with a birthweight below 2kg (AOR = 0.26, 95% CI = 0.14–0.48).

**Table 4:** Binary logistic regression of undernutrition by socio-demographic factors.

Variable	Undernutrition			
	Unadjusted		Adjusted	
	OR (CI)	P-value	AOR (CI)	P-value
**Age of Child (months)**				
0–18 months	**Ref.**			
19–37 months	0.98 (0.89–1.13)	0.38	1.18 (0.97–1.44)	0.10
38+ months	0.78 (0.09–1.12)	0.21	0.81 (0.61–1.09)	0.16
**Sex of Child**				
Male	**Ref.**			
Female	0.67 (0.58–0.71)	**<0.001**	0.71 (0.59–0.85)	**<0.001**
**Birth Weight**				
< 2.5 kg	**Ref.**			
2.5–4.0 kg	0.33 (0.28–0.41)	**<0.001**	0.38 (0.30–0.50)	**<0.001**
4.1 + kg	0.20 (0.11–0.34)	**<0.001**	0.26 (0.14–0.48)	**0.001**
**Birth Type**				
Single birth	**Ref.**			
Multiple births	3.13 (2.47–3.98)	**<0.001**	3.22 (2.11–4.91)	**0.001**
**Birth Order**				
1–4	**Ref.**			
5–8	1.08 (0.96–1.22)	0.20	1.00 (0.79–1.26)	0.97
9 +	0.97 (0.67–1.40)	0.87	0.79 (0.81–1.35)	0.39
**Child Diarrhoea**				
No	**Ref.**			
Yes	1.47 (1.26–1.73)	**<0.001**	1.76 (1.40–2.20)	**<0.001**
**Nutritional Counselling during Pregnancy**				
No	**Ref.**			
Yes	0.89 (0.77–1.02)	0.12	1.06 (0.88–1.027)	0.52
**Number of Children in Household**				
0–4	**Ref.**			
5–9	1.37 (1.11–0.49)	**0.004**	1.21 (0.82–1.77)	0.34
10 +	3.96 (1.54–10.15)	**0.003**	2.20 (0.82–5.89)	0.12
**Maternal age at First Birth**				
11–19	**Ref.**			
20–28	0.84 (0.75–0.94)	**0.003**	0.97 (0.80–1.17)	0.75
29 +	0.78 (0.53–1.14)	0.20	0.64 (0.32–1.24)	0.18
**Education Level**				
Primary	**Ref.**			
No education	1.15 (0.98–1.36)	0.08	1.06 (-0.11–0.26)	0.45
Higher	0.70 (0.57–0.88)	**0.002**	0.82 (0.01–0.42)	**0.036**
**Place of Residence**				
Urban	**Ref.**			
Rural	1.31 (1.12–1.52)	**<0.001**	1.17 (0.94–1.46)	0.16
**Wealth**				
Rich	**Ref.**			
Poor	1.56(1.35–1.81)	**0.001**	1.22 (0.91–1.63)	0.18
Middle	1.09 (0.91–1.31)	0.33	0.94 (0.73–1.21)	0.64
**Marital Status**				
Not married	**Ref.**			
Married	0.21(-0.05–0.48)	0.11	0.67 (0.49–0.93)	**0.03**
**Toilet Facility**				
Improved	**Ref.**			
Unimproved	0.13 (0.12–0.15)	**<0.001**	0.91 (0.71–1.16)	0.46
**Source of Water**				
Improved water	**Ref.**			
Unimproved water	1.16 (1.02–1.31)	**0.02**	0.92 (0.75–1.12)	0.43
**Electricity Access**				
No	**Ref.**			
Yes	0.68 (0.59–0.79)	**<0.001**	1.04 (0.83–1.30)	0.75
**Access to Media**				
No	**Ref.**			
Yes	0.80 (0.70–0.91)	**<0.001**	0.86 (0.70–1.05)	0.16
**Type of Cooking Fuel**				
Improved	**Ref.**			
Unimproved	2.18 (1.38–3.42)	**<0.001**	1.42 (0.83–2.41)	0.20
**Postnatal Visit**				
No	**Ref.**			
Yes	0.97 (0.81–1.16)	0.72	1.05 (0.84–1.31)	0.67
**Current Work Status**				
Not Working	**Ref.**			
Working	0.95(0.82–1.10)	0.50	1.01 (0.78–1.28)	0.96

Note: OR = Odds Ratio; AOR = Adjusted Odds Ratio; Ref = Reference category; p-value considered significant when <0.05

Additional factors associated with undernutrition were identified. Children born from multiple births had 3.22 higher odds of undernutrition compared to those born from a single birth (AOR = 3.22, 95% CI = 2.11–4.91). The presence of diarrhoea was a significant risk factor, with children who had experienced diarrhoea having 1.76 higher odds of undernutrition compared to those who had not (AOR = 1.76, 95% CI = 1.40–2.20).

Maternal education also played a role, with mothers having a higher level of education demonstrating a 0.82 decrease in the risk of undernutrition compared to those with no formal education (AOR = 0.82, 95% CI = 0.01–0.42). Lastly, children under five years of age born to married women had 0.67 lower risk of undernutrition compared to those born to unmarried women (AOR = 0.67, 95% CI = 0.49–0.93).

These findings highlight the significant associations between these factors and the risk of undernutrition among children under five years of age. The results provide important insights into potential intervention strategies and areas of focus to improve nutritional outcomes for this vulnerable population.

## Discussion

This study aimed to examine the combined prevalence of stunting, underweight, and wasting (undernutrition) among children under the age of five years in Benin and the factors that are associated with these undernutrition indicators. A sample consisting of more than 13,000 children aged below five years was analysed, revealing a prevalence rate of 14.95% for stunting, underweight, and/or wasting. This observed percentage is significantly higher compared to the prevalence rate of 12.14% reported in a previous study that employed a similar analytical approach [19]. It is, however, important to highlight that the previous study did not focus on Benin, and instead provided estimates for 31 countries in SSA, encompassing a total population of 127,487 children under five years of age. Subsequent analysis conducted within the specific context of Benin in the present study unveiled the prevalence rates of stunting, wasting, and underweight to be 31.56%, 5.04%, and 16.65% respectively. These findings indicate that in Benin, stunted growth is the most prevalent form of undernutrition among the three indicators, followed by underweight and then wasting. Using the World Health Organisation’s (WHO) cut-off values for undernutrition [34], 31.56% of stunting cases in Benin are classified as “very high,” indicating a significant prevalence of this condition. Additionally, 5.04% of wasting cases are classified as “medium,” representing a moderate prevalence, and 16.65% of underweight cases are classified as “very high,” highlighting a substantial occurrence of underweight among the study population. These findings confirm the hypothesis that undernutrition is a pervasive problem in Benin, highlighting the pressing need for targeted interventions aimed at reducing stunted growth, underweight, and wasting in children under five years of age in the country. The findings of this study may have relevance to other countries in SSA, particularly, those sharing similar socioeconomic and demographic characteristics.

Findings from this study further show that several factors, broadly classified as child and maternal factors, are associated with the prevalence of stunting, underweight and wasting among children under five years of age in Benin. In terms of child-related factors, gender, birth weight, birth type, and a child’s experience of prolonged diarrhoeal episodes were significantly associated with the prevalence of stunting, underweight and wasting among children under five years of age in Benin. Concerning maternal factors, mothers’ educational level and marital status were significantly associated with the prevalence of stunting, underweight and wasting among children under five years of age in the country. Together, these findings more specifically indicate that the likelihood of an under-five child experiencing stunting, underweight, and wasting is significantly associated with the male gender, lower birth weight, childhood experience of prolonged diarrhoeal episodes, lower levels of maternal education, and marriage.

The finding that females under the age of five years had decreased odds of stunting, underweight, and wasting compared to males under five years of age is consistent with several previous studies conducted in SSA and other regions of the world [19, 35, 36]. This observation may be attributed to socio-cultural beliefs that influence food allocation practices, whereby male children are perceived to require more calories than female children [37, 38]. These beliefs may lead to unequal food distribution and portion sizes within households which may, in turn, result in potential overeating or overweight among male children [37, 38]. Conversely, female children under five years of age may experience indirect promotion of undernutrition due to parental feeding styles [39]. The results suggest that efforts to address gender inequalities in food allocation and distribution may have a positive impact on reducing undernutrition in both male and female children under five years of age in Benin. There is also a need for utilising qualitative approaches to investigate the socio-cultural factors that influence parental feeding practices specifically towards male and female children under the age of five years in Benin. In-depth qualitative investigations can provide valuable insights into the complex interplay between cultural norms, gender roles, and dietary patterns that may shape parental attitudes and behaviours related to child undernutrition. Such information is crucial for developing context-specific interventions that address the root causes of undernutrition and promote equitable feeding practices for all children under the age of five years, regardless of gender.

Studies show that low birth weight is associated with childhood undernutrition [40, 41]. In line with previous evidence [40, 41], findings in this present study showed that children under five years of age with a birth weight of 4 kg or more had a lower risk of stunting, underweight and wasting compared to those with lower birth weights. This implies that children with low birth weights have a higher risk of continuous undernutrition in the early years of their childhood if no intervention is implemented. This highlights the importance of early interventions to prevent continuous undernutrition among children with low birth weights. However, this result should be taken cautiously as the results showed that those with birth weights lower than 2.5 kg had a lower prevalence of stunting, underweight and wasting than those whose birth weights were between 2.5 and 4 kg. Further research is needed to explore the potential underlying factors and mechanisms that might account for this unexpected finding.

Consistent with outcomes in previous studies [42, 43], the present study found that children under five years of age who were a result of multiple births and those who experienced prolonged episodes of acute diarrhoea had a significant risk of stunting, underweight and wasting in Benin. A logical explanation for these findings is that children who are a result of multiple births could be facing competition for food whereas those with prolonged episodes of acute diarrhoea may be experiencing acute dehydration and loss of nutrients leading to their under-nutrition status. A study published in the Lancet Global Health similarly suggests that the burden of diarrhoea among children under 5 years old is multifaceted and accounts for the risk of childhood growth faltering [44]. It is therefore important for the government of Benin, international health organisations, as well as non-governmental organisations in Benin, to support the provision of food incentives to mothers with multiple births and those whose under-five children experience diarrhoeal episodes to help minimise the risks of child undernutrition in the country [45].

Findings of this study indicate that children under the age of five years in Benin, who were born to mothers with higher levels of education, had a lower risk of experiencing stunting, underweight, and wasting compared to those whose mothers had lower levels of education. This result suggests that maternal education plays a significant role in shaping the nutritional status and overall well-being of young children in Benin. Based on the results, it is reasonable to suggest that mothers in Benin who had received some level of formal education were more likely to have been exposed to information about the benefits of childhood nutrition, particularly through print media sources. This exposure may have contributed to greater awareness and understanding of optimal feeding practices. Consequently, it is plausible that these educated mothers had a more favourable feeding experience with their children, leading to improved nutritional outcomes [45]. This finding highlights the importance of promoting education for girls and women in Benin, as well as providing education and support to mothers with lower levels of education to improve their child-feeding practices and overall nutritional status. Such interventions could involve the provision of nutrition education and counselling services, as well as efforts to increase access to educational resources and training programs for mothers in underserved communities.

One of the understudied variables in childhood nutrition is the impact of marriage on the risk of stunting, underweight, and wasting among children under the age of five years. In this study, marriage was found as a protective factor against the risk of stunting, underweight, and wasting among children under five years in Benin. The finding in the current study corroborates a study in Bangladesh that indicated that the marital status of mothers can affect child nutrition, primarily from a resource-effect perspective [46]. Thus, married women are more likely than unmarried women to be predisposed to higher levels of household income which may increase their food purchasing power, and in effect, improve their children’s nutritional status.

Based on the findings, we strongly recommend comprehensive support from both governmental and non-governmental organisations to address the significant risk of stunting, underweight, and wasting among specific subgroups of children under five years in Benin. Targeted interventions should prioritise the following groups: female children, children with low birth weights, children experiencing prolonged diarrheal episodes, children born as multiples (more than one child during delivery), mothers with limited education, and single mothers. There is potential to draw lessons from the successful experience of Thailand in controlling undernutrition for over two decades. By studying and adapting the strategies employed by Thailand, Benin can enhance its efforts to combat undernutrition effectively [47]. Decades ago, Thailand was among the developing countries with endemic childhood undernutrition [47]. A Poverty Alleviation Plan was developed in the country aimed at achieving basic minimum needs (BMNs), including a reduction in child malnutrition [47]. Community-based programmes comprising multifaceted nutrition aid services administered by mass volunteers were implemented in the country, including the provision of milk and lunch in schools. These programmes have yielded positive results and led to a decline in underweight among pre-schoolers from 51% in 1980 to 20% in 1990 and then to below 10% in 2006 [47]. The government of Benin, non-governmental organisations, and volunteers can implement similar policies or programmes for children under five years with an increased focus on children at significant risk of undernutrition as reported in this study, such as those of female gender, low birthweight, products of multiple births, and those belonging to uneducated mothers [41]. However, it is crucial to ensure that these programmes are regulated and guided by a team of qualified nutritionists who can provide expert advice on appropriate feeding practices. While addressing the undernutrition problem, it is important to avoid the risk of inadvertently promoting overnutrition or other nutritional imbalances.

### Strengths and limitations

The present study makes a significant contribution to the existing literature by identifying nutrition-related factors specific to Benin, a country with a disproportionately high prevalence of stunting, underweight, and wasting among children under five years of age. While a previous study has focused on nutritional indicators in Sub-Saharan Africa (SSA) [19], the present study reports several factors associated with childhood stunting, underweight, and wasting that were not captured in the previous study, notably: number of delivered children, child diarrhoea, mothers who used surface water, mothers with unimproved cooking fuel, and nutritional counselling during pregnancy. Moreover, the present study is based on nationally representative data of under-5 children in Benin, making the findings reliable.

Despite the strengths, this study has some limitations that need to be acknowledged. Firstly, the cross-sectional design employed in the Demographic and Health Survey (DHS) limits the establishment of causality in the findings. Additionally, due to the cross-sectional nature of the data, the findings may not be generalisable to other regions. Furthermore, the self-report method used in the survey is susceptible to recall and social desirability bias. For instance, recalling events in five years preceding the survey may be associated with errors in the parametric estimations, which could have potentially affected the findings. Additionally, diarrhoea in children was measured in two weeks preceding the survey, which is likely to result in weight loss in children under 5, is likely reversible, and may not lead to a sustained underweight effect. Although this study focused mainly on socio-demographic determinants of health, cultural factors may also contribute to variations in stunting, underweight, and wasting among children under five years of age. Finally, the study combined data from three different indicators, making the interpretation of the findings quite complicated.

### Conclusions, recommendations, and future direction

The eradication of childhood undernutrition is vital in achieving the Sustainable Development Goal (SDG) 2, which seeks to eliminate hunger, promote sustainable agriculture, and improve nutrition [48]. In light of this, tackling the undernutrition challenge among under-five children in Benin is crucial in advancing the SDG agenda [45]. Consequently, findings from this present study have significant implications for global development efforts, underscoring the importance of stakeholders taking appropriate measures to address the global undernutrition problem.

In conclusion, the current study highlights that undernutrition among children under five years of age is a significant issue in Benin. The study found a prevalence rate of 14.95% for stunting, underweight, and/or wasting, indicating a substantial burden of undernutrition within this population. This finding underscores the urgent need for targeted interventions and strategies to address and mitigate the prevalence of undernutrition in Benin, aiming to improve the nutritional status and overall well-being of young children [49, 50]. In addition, the study concludes that several factors are associated with childhood undernutrition in Benin. These factors include low birthweight, multiple births, female gender, prolonged diarrhoeal episodes, low maternal education level, and marital status. These findings emphasise the importance of implementing effective strategies to improve antenatal and postnatal care in order to address these risk factors.

Targeted childhood nutrition programmes should be developed and implemented to specifically address the nutritional needs of children in Benin. By targeting the key factors highlighted in this study, Benin can work towards reducing the prevalence of undernutrition and improving the overall health and well-being of children in the country.

The role of socio-cultural factors in childhood undernutrition in the country may also be significant [51], and future studies should explore this further. Capturing the experiences and perspectives of mothers regarding cultural feeding practices could shed light on how feeding behaviour or styles may contribute to undernutrition [51]. Additionally, efforts should be made to develop a single undernutrition indicator for use in prospective Benin Demographic and Health Surveys.
